# Modulation of dopamine release by α7-type nicotinic acetylcholine receptors

**DOI:** 10.1186/1471-2202-14-S1-P155

**Published:** 2013-07-08

**Authors:** Reinoud Maex, Vladimir Grinevich, Evgeny Budygin, Merouane Bencherif, Boris Gutkin

**Affiliations:** 1 Department of Cognitive Sciences, École Normale Supérieure, Paris 75005, France; 2 PET Center, Wake Forest Baptist Medical Center, Winston-Salem, NC 27157, USA; 3 Department of Neurobiology & Anatomy, Wake Forest Baptist Medical Center, Winston-Salem, NC 27157, USA; 4 Targacept Inc., Winston-Salem, NC 27101, USA

## 

Disentangling receptor kinetics and network dynamics is a prerequisite for understanding and predicting the systemic effects of pharmacological compounds. These effects may result from a complex balance between activation and desensitization of various receptor subtypes that can be located on excitatory or inhibitory neurons or their afferent fibres. We therefore combined receptor and network modelling [1] to explain the suppressive effect that TC-7020 [2,3], a partial agonist of α7-type nicotinic acetylcholine receptors (nAChRs), was observed to exert on the release of dopamine (DA) in the nucleus accumbens of anesthetized rats. As nAChRs are ion channels permeating Na^+ ^and Ca^2+^, and as i.v. injection of nicotine itself enhanced DA release, partial agonists would rather be expected to enhance DA release as well.

Using the model, we evaluated several potential effects of TC-7020 on DA-ergic and GABA-ergic neurons in the ventral tegmental area (VTA) and its glutamatergic afferents from neocortex. Potential effects of TC-7020 included on the one hand a reduced nAChR channel conductance on DA neurons through competitive or non-competitive inhibition, or through receptor desensitization. On the other hand, enhancing the nAChR conductance on GABA-neurons (through direct activation of nAChRs, or through priming of their response to background ACh [4]) could have produced, through inhibition of DA neurons, a similar suppressive effect on DA release. The model showed that rapid desensitization of the α7 nAChRs on DA neurons explained both the sign and time-course of the voltammetric DA response, provided that a background cholinergic tone was present that contributed, by activating α7 nAChRs without desensitizing them, to the baseline level of DA release.

Another surprising finding of the voltammetric DA recordings was that PNU-120596, a positive allosteric modulator of α7 nAChRs, prevented and abolished the effect of TC-7020 on DA release, instead of amplifying it. The model proposes two possible but contrasting mechanisms: a well-established relieve of α7 nAChRs from the weak state of desensitization into which they were brought by TC-7020, or, in contrast, the induction of a strong desensitization in α7 nAChRs due to channel over-excitation by background ACh under the amplifying effect of PNU-120596 [5].

These results show that tonic levels of neurotransmitters and neuromodulators like acetylcholine and dopamine may profoundly affect the dynamics, and hence the systemic effect, of pharmacological compounds. As these background levels may be altered in in-vitro preparations, circuit modelling may become an indispensible tool in drug development.
